# Chronic prostatitis: Current concepts

**DOI:** 10.4103/0970-1591.38598

**Published:** 2008

**Authors:** Ram Vaidyanathan, Vibhash C. Mishra

**Affiliations:** Department of Urology, Wexham Park Hospital, Slough, UK

**Keywords:** Chronic pelvic pain syndrome, chronic prostatitis, prostate, prostatitis

## Abstract

**Purpose:**

Chronic prostatitis (CP) is a common condition. It causes significant suffering to the patients and constitutes a sizeable workload for the urologists. The purpose of this review is to describe the currently accepted concepts regarding the aspects of CP.

**Materials and Methods:**

Relevant papers on the epidemiology, etiology, diagnosis, evaluation and management of CP were identified through a search of MEDLINE using text terms “prostatitis”, “chronic prostatitis” and “chronic pelvic pain syndrome”. The list of articles thus obtained was supplemented by manual search of bibliographies of the identified articles and also by exploring the MEDLINE option “Related Articles”.

**Results:**

The salient points of the relevant articles on each aspect of CP have been summarized in the form of a non-systematic narrative review.

**Conclusion:**

Chronic prostatitis is caused by a variety of infective and non-infective factors and is characterized by a rather long remitting and relapsing clinical course. The diagnosis is based on symptoms comprising pain and nonspecific urinary and/or ejaculatory disturbances and microbiological tests to localize bacteria and/or leucocytes in segmented urinary tract specimens. The contemporary classification was proposed by the National Institutes of Health/National Institute of Diabetes Digestive Kidney Diseases (NIH/NIDDK). National Institutes of Health - Chronic Prostatitis Symptom Index (NIH-CPSI) is the patient evaluation tool used extensively in clinical practice and research. Management should be individualized, multimodal and of an appropriate duration.

## INTRODUCTION

Chronic prostatitis (CP) is a common, yet poorly understood condition. It spells misery not only for the patients who have to suffer, but also for the urologists looking after them. They often find themselves running out of ideas when faced with this difficult problem, as there are few effective treatment options available at their disposal. Research in this field in the last few years has centered on the generation of a new classification system and a symptom score to evaluate patients with. It is expected that better categorization of patients will result in more individualized use of the existing treatment modalities, some of which have recently been tested in a randomized controlled setting. The purpose of this update is to familiarize the reader with the currently accepted concepts in a continuously evolving area.

The term chronic prostatitis should be considered a misnomer in that the entity may not necessarily be inflammatory or exclusively prostatic in origin. Bearing in mind this caveat, CP can be defined as a clinical syndrome characterized by pain in the perineum, pelvis, suprapubic area or the external genitalia, with a variable degree of voiding and/or ejaculatory disturbance.[1]

## EPIDEMIOLOGY

Prostatitis is a significant health problem with prevalence rates of 11-16%.[2,3] More than 2 million consultations for prostatitis are required every year in the United States[4] and each Canadian urologist treats about 262 such patients every year.[5] Prostatitis is the most common reason for men under 50 to consult a urologist[4] and indeed it generates more physician visits than benign prostatic hyperplasia or prostate cancer in the United States.[6] It has a significant impact on the quality of life (QoL) comparable to active Crohn's disease or a recent myocardial infarction.[7] Up to 50% of men may be affected by it at some stage of their lives.[8,9]

## CLASSIFICATION

Rather than being a single entity, prostatitis encompasses a spectrum of closely related symptom complexes of varied etiopathogenesis. Any classification of this condition should recognize this fact. Drach *et al.* were the first to use a systematic approach to the diagnosis and management of patients with symptoms of prostatitis, based on the microscopic examination and cultures of segmented urogenital tract specimens.[10]

A workshop on prostatitis sponsored by National Institutes of Health/National Institute of Diabetes, Digestive and Kidney diseases (NIH/NIDDK)[11] in 1995 agreed on the standard contemporary classification system, which is Shown in Table 1.

**Table 1:** The NIH prostatitis classification

Category	Designation	Status of infection
I	Acute bacterial prostatitis	Acute infection of prostate
II	Chronic bacterial prostatitis	Recurrent infection of prostate
III	Chronic non-bacterial prostatitis/Chronic pelvic pain syndrome (CPPS)	No demonstrable infection
IIIA	Inflammatory	WBC in semen/EPS/post-prostatic massage urine
IIIB	Non-inflammatory	No WBC in semen/EPS/post-prostatic massage urine
IV	Asymptomatic inflammatory prostatitis	Asymptomatic

WBC - white blood cell, EPS - expressed prostatic secretion

## AETIOPATHOGENESIS

A myriad of etiological factors – some not even involving the prostate gland – have been postulated. The initiator of the inflammatory process could be a local infection, chemical irritation, dysfunctional voiding, intraductal reflux, neuromuscular disturbances or an immunological process. *There may be an etiological link between Category III prostatitis and interstitial cystitis (IC). The pathogenesis is not entirely certain, but the following may be a possible mechanism*. Regardless of the triggering factor, the resultant inflammatory process causes tissue edema and increased intra-prostatic pressure leading to local hypoxia and varied mediator-induced tissue damage. This leads to altered neurotransmission in sensory nerve fibers thereby resulting in the pain and other symptoms associated with the condition.

### Infection

Category I and II prostatitis are caused by bacterial infection. The role of infection in Category III prostatitis is not established although cryptic, non-culturable organisms may be responsible.

Bacteria can be isolated preferentially from an expressed prostatic secretion (EPS) or a post-prostatic massage urine specimen rather than from the mid-stream urine (MSU) sample[12] or can be demonstrated on the prostatic biopsy specimen.[13,14] Common pathogens are aerobic gram-negative bacteria and gram-positive cocci including enterococci and staphylococci. Several other anterior urethral commensals can also cause prostatitis under special circumstances.[8]

In Category III prostatitis, although absence of bacterial infection is inherent in the definition, numerous investigators have proposed possible infection with atypical or fastidious organisms.[13,15] Identification of bacterial DNA[16] and clinical improvement sometimes seen with antibiotic treatment[17] in this group provide justification for the trial of antibiotic treatment in these patients despite the lack of laboratory-proven infection.

Lack of uniformity in response to antibacterial treatment and the inability to consistently isolate any pathogenic organisms in the appropriate specimens in addition to the difficulties in determining the respective roles of commensal versus virulent organisms have led to the search for other etiological factors.

### Dysfunctional high-pressure voiding

The NIH classification emphasized genitourinary pain as the main characteristic of CP. Pain and the lower urinary tract symptoms associated with CP are by no means specific to the condition and may be caused by either an anatomic or physiological cause of lower urinary tract obstruction such as bladder neck stenosis,[18] urethral stricture, detrussor sphincter dyssynergia[19] or a dysfunctional voiding pattern.[20] Video pressure flow studies with synchronous electromyography of external sphincter have demonstrated decreased flow rate and increased maximal urethral closure pressure. This was observed in both Category IIIA and IIIB patients.[21,22] This explains why some of these patients respond favorably to the use of α-blockers.[21]

### Intraprostatic ductal reflux

Crystallographic analysis of prostatic calculi has shown them to be made exclusively of urinary constituents[23] and high levels of urate and creatinine have been found in EPS.[24] This suggests intraprostatic ductal reflux as another possible etiological factor in CP. As a result of urine reflux, chronic inflammation and tissue edema may lead to voiding disturbances with further reflux of urine perpetuating a vicious cycle.

### Autoimmunity

Several authors[25,26] have suggested the inflammation in CP to be caused by an autoimmune process or induced by an unknown antigen. Elevated levels of pro-inflammatory cytokines[25] are present in seminal plasma in patients with Category III prostatitis. Shoskes *et al.*,[27] also found low levels of the anti-inflammatory cytokine IL-10 in men with prostatitis.

Immune response in CP seems to be T-cell mediated with cytotoxic (CD8) T cells predominating over helper (CD4) T cells.[28] COX 2 is undetectable in normal tissues but is over-expressed in tissues with inflammation and possibly upregulated by inflammatory cytokines IL-1B and TNFα. NSAIDs blocking COX 1 and 2 have been successfully used in the treatment of CP.

### Interstitial cystitis

There are striking similarities in the clinical presentation of CP and IC. Occult infection, “leaky epithelium”, neurogenic inflammation, mast cell activation and autoimmunity have been implicated in the etiology of both conditions. Mastocytosis and elevated urinary histamine levels in patients with CP have been reported and Parsons potassium sensitivity test has been shown to be positive in over 80% of Category IIIA prostatitis. A trial of Pentosan polysulfate in Category IIIA prostatitis showed a significant difference in the symptom scores and there is a case for more extensive evaluation of the role of agents to restore the epithelial integrity.[29]

### Neuromuscular problem

One of the several hypotheses suggests that chronic pain in CP is neuropathic in character and there is a neuromuscular etiology for both Type IIIA and IIIB prostatitis. Type IIIB prostatitis may be a manifestation of reflex sympathetic dystrophy.[30]

Neurogenic inflammation is a process by which nerves may secrete mediators producing local inflammation and /or hyperalgesia. This role of neurogenic inflammation in Category III prostatitis has not been extensively studied. However, symptomatic improvement with intravesical instillation of substances like dimethylsulfoxide[31] suggests that it may play a role in the symptomatic exacerbation in some patients.

## CLINICAL PRESENTATION AND EVALUATION

As mentioned at the outset, the symptoms of CP are non-specific and there is great overlap between this condition and other causes of lower urinary tract dysfunction. By definition, the symptoms must have been present for at least three months, though in practice, the patients have usually had them for several years. Waxing and waning of symptoms is a common occurrence.

Chronic prostatitis is essentially a diagnosis of exclusion. Thus the evaluation should aim at excluding other possible causes for the patients' symptoms such as benign prostatic hyperplasia, urethral stricture, urinary infection etc. and a recommended workup is shown in Table 2.

**Table 2:** Evaluation of the patient presenting with symptoms suggestive of chronic prostatitis

All patients
History *including NIH-CPSI*
Physical examination including DRE
Urinalysis and Urine culture - midstream
Flow rate
Residual urine determination
Selected patients
IPSS Questionnaire
Urine cytology
Urethral swab
Semen culture
PSA
Cystoscopy
Urodynamic studies
Prostatic imaging

Once these competing conditions have been excluded, the evaluation should be carried out using the National Institutes of Health – Chronic Prostatitis Symptom Index (NIH-CPSI) [Table 3], which is a validated tool generated from the NIH/ NIDDK workshop and recommended for use in clinical practice as well as research.[11]

**Table 3:** NIH- Chronic Prostatitis Symptom Index (NIH-CPSI)

**Pain or discomfort**
1. In the last week, have your experienced any pain or discomfort in the following areas
Yes No
a. Area between rectum and testicles (perineum) 1 0
b. Testicle 1 0
c. Tip of penis not related to urination 1 0
d. Below waist in pubic or bladder area 1 0
2. In the last week, have your experienced:
a. pain or burning during urination 1 0
b. pain or discomfort during or after ejaculation 1 0
3. How often have you had pain or discomfort in any of these areas over the last week?
0 Never
1 Rarely
2 Sometimes
3 Oftern
4 Usually
5 Always
4. Which number best describes your average pain or discomfort on the days that you had it, over the last week?
0 1 2 3 4 5 6 7 8 9 10
**Urination**
5. How often have you had a sensation of not emptying your bladder completely after you finished urinating over the last week?
0 None at all
1 Less than 1 time in 5
2 Less than half the time
3 About half the time
4 More than half the time
5 Almost always
6. How often have you had to urinate again less than two hours after you finished urinating, over the last week?
0 None at all
1 Less than 1 time in 5
2 Less than half the time
3 About half the time
4 More than half the time
5 Almost always
**Impact of symptoms**
7. How much have your symptoms kept you from doing the kinds of things you would usually do over the last week?
0 None 1 Only a little 2 Some 3 A lot
8. How much did you think about your symptoms over the last week?
0 None 1 Only a little 2 Some 3 A lot
Quality of life
9. If you were to spend the rest of your life with your symptoms, just the way they have been during the last week, how would you feel about it?
0 Delighted
1 Pleased
2 Mostly satisfied
3 Mixed (about equally satisfied and dissatisfied)
4 Mostly dissatisfied
5 Unhappy
6 Terrible
**Scoring the NIH-CPSI prostatitis symptom index domain**
Pain:
Total of items 1a, 1b, 1c, 1d, 2a, 2b, 3 and 4 =
Urinary Symptoms:
Total of items 5 and 6 =
Quality of life impact:
Total of items, 7, 8 and 9 =

The NIH-CPSI examines the three main domains of prostatitis namely pain, urinary symptoms and QoL. It is a nine-item questionnaire that is simple, easy and quick to administer and should be used to establish the patients' baseline bother, which should inform the requirement for treatment, to stratify patients on the basis of their predominant symptoms and accordingly to select the best treatment and to monitor the response to treatment.

## DIAGNOSIS

The diagnosis is based on symptomatology and localization of organisms and/or leucocytes in segmented urogenital tract specimens. Of all evaluation methods, the gold standard is the four glass test proposed by Meares and Stamey.[32] It involves obtaining the following specimens for microscopy and culture - the first voided 5-10 ml urine (VB1), midstream urine (VB2), pure prostatic secretion expressed by prostatic massage (EPS) and the first voided 5-10 ml urine after prostatic massage (VB3). *The findings of organism(s) with or without WBCs, WBCs without organisms and neither WBCs nor organisms in EPS and/or VB3 indicate a diagnosis of Category II, IIIA and IIIB prostatitis respectively.*

A less tedious, time-consuming and expensive but almost equally effective modification of the four glass test, the pre- and post-massage test (PMPT), has been proposed by Nickel.[33] The PMPT involves microscopy and culture of pre- and post-prostatic massage urine sample with a positive post-massage sample indicating the possibility of CP.

## MANAGEMENT

As is evident from the preceding discussion, CP is not a single disease entity and different factors may be involved not only in different patients but even in an individual patient. Thus the treatment should be individualized. To date there are few data to support rational therapeutic decisions for the management of CP. Studies providing unequivocal evidence of efficacy of any particular modality of treatment are few and far between and therefore, recommendations are commonly based on clinical and anecdotal experience of experts. Symptom control rather than eradication seems a more realistic objective and to be effective towards that goal, the management should be multimodal, of an appropriate duration and incremental in nature.

We have briefly outlined the investigated treatment options based on the material available in literature.

### Antimicrobial agents

Bacteria such as *E. Coli, Enterococci, Pseudomonas, Klebsiella* and *Proteus* have been implicated in Type I and Type II prostatitis. Moreover, even in Type IIIA prostatitis, there may be a role of *Chlamydia, Ureaplasma* and other fastidious organisms or viruses. Results of treatment are generally better for CP due to *E. coli* and other enterobactericeae than that caused by *Pseudomonas* and *Enterococci*.[9] The commonly used antibiotics include Quinolones, Azithromycin, Tetracycline, Co-trimoxazole and amoxicillin. Use of a quinolone for at least a month is the most popular regime and seems to be better than co-trimoxazole.[34] It is usually effective in culture-proven Category II patients, but used quite frequently even in the absence of a positive culture.

### Anti-inflammatory agents

The theory behind the use of NSAIDs is that they reduce pain, inhibit activation and function of neutrophils and reduce edema. The primary symptoms of chronic prostatitis are perineal, lower abdominal, testicular, penile and ejaculatory pain. Relief of the pain and discomfort associated with CP is the primary goal of therapy. NSAID therapy is one of the most common treatments for CP. However, hard evidence for NSAID therapy has been provided by only one randomized controlled study, which showed a higher percentage of patients with a 25% or six-point improvement in total NIH/CPSI score with 50 mg rofecoxib for six weeks compared to placebo.[35]

### Muscle relaxants

Diazepam and baclofen can be used in Category IIIB prostatitis when sphincter dyssynergia or pelvic floor/perineal muscle spasm is confirmed. The evidence for this is rather old[36] and the role of these agents has not been re-evaluated subsequently.

### Alpha-blockers

The rationale for using alpha-blockers is provided by the significant overlap of obstructive and irritative symptoms between prostatitis and benign prostatic hyperplasia. Therefore, by derivation a drug that helps the symptoms of one condition should also be effective for the other. Moreover, Barbalias observed increased maximum urethral closure pressure caused by hypertonia of the prostatic urethra from adrenergic over-activity in CP, resulting in dysfunctional voiding, intraductal reflux and nonspecific inflammation.[37] A recent systematic review of the literature concluded that alpha-blockers administered for at least three months may have a useful role in the treatment of severely symptomatic, treatment naïve patients with Type III prostatitis who have particularly high scores in the NIH-CPSI urinary domain.[38]

### Alpha reductase inhibitors

5 alpha reductase inhibitors (5 ARIs) are well known to reduce lower urinary tract symptoms by decreasing the gland size. They may also reduce the intraprostatic reflux and thereby the inflammatory process.[39]

### Phytotherapy

There are two phytotherapeutic agents that have been evaluated for use in CP: quercetin and cernilton.[40,41] Quercetin is an anti-oxidant and nitric oxide inhibitor. Cernilton may be associated with cyclooxygenase inhibition or relaxation of smooth muscles. Both agents have been shown to produce significant symptomatic improvement though the exact mechanism of action is not well understood.

### Prostatic massage

The benefits of prostatic massage are believed to be derived from a combination of several factors including expression of inspissated prostatic secretions, relief of pelvic muscle spasm, physical disruption of any protective biofilm, improved circulation and thus penetration of antibiotics.[42] The only randomized controlled trial failed to show any benefit from prostatic massage.[43] However, there are case series and anecdotal reports suggesting that bi- or tri-weekly massage for six to 12 weeks used alongside antibiotics possibly provides some symptomatic relief to a quarter to a third of patients with CP.[33,44]

### Thermotherapy

A Cochraine Review evaluated five studies of different types of heat treatments [either transrectal microwave hyperthermia (TRMH) or transurethral microwave thermotherapy (TUMT)] for Type III prostatitis and showed significant improvements in symptoms with heat treatment compared with sham treatment.[45]

### Electromagnetic therapy

In a small randomized controlled trial, Rowe *et al.*, demonstrated a statistically significant difference in NIH-CPSI scores of men with Type III prostatitis treated with pelvic electromagnetic therapy compared to placebo.[46]

The following general management recommendations have been made on the basis of evidence currently available in the literature.[33]

Type I prostatitis is effectively treated with antibiotics and Type IV does not require treatment.

Patients with Type II should be treated with six to 12 weeks of antibiotics, which should be combined with prostatic massage for refractory or relapsing cases, *although there is a lack of hard evidence for much benefit from massage in this situation*.

Type IIIA prostatitis should first be treated with antibiotics and prostatic massage. *Once again the evidence for the latter is soft*. Alpha-blockers should be used for patients with a particularly high score in the NIH-CPSI urinary domain. For refractory cases, anti-inflammatories, 5ARIs, phytotherapy or TUMT may be considered.

Patients with Type IIIB prostatitis are the most difficult to treat. Alpha-blockers, analgesics, muscle relaxants and tricyclic antidepressants used concurrently may provide amelioration of symptoms.

## CONCLUSION

In summary, CP is a common problem affecting relatively younger men. It is characterized by a variable mix of pain, urinary and ejaculatory symptoms. The etiology is multifactorial. Diagnosis is based on symptoms and localization tests, which also provide the basis for classification. There are multiple options for treatment, which should be used according to the individual merit of each case to achieve what may often be only symptom control rather than eradication.

## References

[1] Krieger JN, Egan KJ, Ross SO, Jacobs R, Berger RE (1996). Chronic pelvic pains represent the most prominent urogenital symptoms of ‘Chronic prostatitis’. Urology.

[2] Roberts RO, Lieber MM, Rhodes T, Girman CJ, Bostwick DJ, Jacobsen SJ (1998). Prevalence of a physician-assigned diagnosis of prostatitis: The Olmstead County study of urinary symptoms and health status among men. Urology.

[3] Collins MM, Meigs JB, Barry MJ, Walker Corkery E, Giovannucci E, Kawachi I (2002). Prevalence and correlates of prostatitis in the health professionals follow-up study cohort. J Urol.

[4] Collins MM, Stafford RS, O'Leary MP, Barry MJ (1998). How common is prostatitis? A national survey of physician visits. J Urol.

[5] Nickel JC, Nigro M, Valiquette L, Anderson P, Patrick A, Mahoney J (1998). Diagnosis and treatment of prostatitis in Canada. Urology.

[6] Roberts RO, Lieber MM, Bostwick DG, Jacobsen SJ (1997). A review of clinical and pathological prostatitis syndromes. Urology.

[7] Wenninger K, Heiman J, Rothman I, Berguis JP, Berger BE (1996). Sickness impact on chronic nonbacterial prostatitis and its correlates. J Urol.

[8] Lobel B, Rodriguez A (2003). Chronic prostatitis: What we know, what we don't know and what we should do!. World J Urol.

[9] Stamey TA (1981). Periurethral or perineal bacteria in urinary tract infections?. JAMA.

[10] Drach GW, Fair WR, Meares EM (1978). Classification of benign diseases associated with prostatic pain: Prostatitis or prostatodynia?. J Urol.

[11] Litwin MS, McNaughton-Collins M, Fowler FJ, Nickel JC, Calhoun EA, Pontari MA (1999). The NIH Chronic Prostatitis Symptom Index (NIH-CPSI): Development and validation of a new outcomes measure. J Urol.

[12] Nickel JC, Bruce AW, Reid G, Krane RJ, Siroky MB (1994). Pathogenesis, diagnosis and treatment of the prostatitis syndromes. Clinical urology.

[13] Berger RE, Krieger JN, Rothman I, Muller CH, Hillier SL (1997). Bacteria in the prostate tissue of men with idiopathic prostatic inflammation. J Urol.

[14] Nickel JC, Costerton JW (1993). Bacterial localization in antibiotic-refractory chronic bacterial prostatitis. Prostate.

[15] Jarvi K, Wang W, B (2001). Identification of unusual bacteria in the semen and urine of men with NIH type III prostatitis. J Urol.

[16] Shoskes DA, Shahed AR (1970). Detection of bacterial signal by 16S rRNA polymerase chain reaction in expressed prostatic secretions predicts response to antibiotic therapy in men with chronic pelvic pain syndrome. Tech Urol.

[17] Nickel JC, Downey J, Johnston B, Clark J, Canadian Prostatitis Research Group (2001). Predictors of patients' response to antibiotic therapy for the chronic prostatitis/ chronic pelvic pain syndrome: A prospective multicenter clinical trial. J Urol.

[18] Kaplan SA, Te AE, Jacobs BZ (1994). Urodynamic evidence of vesical neck obstruction in men with misdiagnosed chronic nonbacterial prostatitis and the therapeutic role of endoscopic incision of the bladder and neck. J Urol.

[19] Kaplan SA, Santarosa RP, D'Alisera PM, Fay BJ, Ikeguchi EF, Hendricks J (1997). Pseudodyssynergia misdiagnosed as chronic nonbacterial prostatitis and the role of biofeedback as a therapeutic option. J Urol.

[20] Hellstrom WJ, Schmidt RA, Lue TF, Tanagho EA (1987). Neuromuscular dysfunction in nonbacterial prostatitis. Urology.

[21] Barbalias GA, Nikiforidis G, Liatsokos EN (1998). Alpha-blockers for treatment of chronic prostatitis in combination with antibiotics. J Urol.

[22] Barbalias GA (1990). Prostatodynia or painful male urethral syndrome?. Urology.

[23] Kirby RS, Lowe D, Bultitude MI, Shuttleworth KE (1982). Intraprostatic urinary reflux:an etiological factor in abacterial prostatitis. Br J Urol.

[24] Persson BE, Ronquist G (1996). Evidence for a mechanistic association between nonbacterial prostatitis and levels of urate and creatinine in expressed prostatic secretions. J Urol.

[25] Alexander RB, Brady F, Ponniah S (1997). Autoimmune prostatitis: Evidence of T cell reactivity with normal prostatic proteins in men with history of prostatitis. Urology.

[26] Doble A, Walker MM, Harris JR, Taylor-Robinson D, Witherow RO (1990). Intraprostatic antibody deposition in chronic abacterial prostatitis. Br J Urol.

[27] Shoskes DA, Albakri Q, Thomas K, Cook D (2002). Cytokine polymorphism in men with chronic prostatitis/ chronic pelvic pain syndrome:association with diagnosis and treatment response. J Urol.

[28] John H, Barghorn A, Funke G, Sulser T, Hailemariam S, Hauri D (2001). Noninflammatory chronic pelvic pain syndrome: Immunological study in blood, ejaculate and prostate tissue. Eur Urol.

[29] Eisenberg ER, Moldwin RM (2003). Etiology: Where does prostatitis stop and interstitial cystitis begin?. World J Urol.

[30] Egan KJ, Krieger JL (1997). Chronic abacterial prostatitis: A urological chronic pain syndrome?. Pain.

[31] Shirley SW, Stewart BH, Mirelman S (1978). Dimethyl sulfoxide in the treatment of inflammatory genitourinary disorders. Urology.

[32] Meares EM, Stamey TA (1968). Bacteriologic localization patterns in bacterial prostatitis and urethritis. Invest Urol.

[33] Nickel JC (2000). Prostatitis: Lessons from the 20th century. BJU Int.

[34] Weidner W, Shiefer HG, Brahler E (1991). Refractory chronic bacterial prostatitis: A re-evaluation of ciprofloxacin treatment after median follow up of 30 months. J Urol.

[35] Nickel JC, Pontari M, Moon T, Gittelman M, Malek G, Farrington J (2003). A randomized, placebo controlled, multicenter study to evaluate the safety and efficacy of rofecoxib in the treatment of chronic nonbacterial prostatitis. J Urol.

[36] Osborne DE, George NJ, Rao PN (1981). Prostatodynia: Physiological characteristics and rational management with muscle relaxants. Br J Urol.

[37] Barbalias GA (2003). Why alpha-blockers in prostatitis?. Eur Urol.

[38] Mishra VC, Browne J, Emberton M (2007). Role of alpha-blockers in type III prostatitis: A systematic review of the literature. J Urol.

[39] Holm M, Meyhoff HH (1997). Chronic prostatitis pain: A new treatment option with finasteride?. Scand J Urol Nephrol.

[40] Buck AC, Rees RW, Ebeling L (1989). Treatment of chronic prostatitis and prostatodynia with pollen extract. Br J Urol.

[41] Shoskes DA, Zeitlin SI, Shahed A, Rajfer J (1999). Quercetin in men with category III chronic prostatitis: A preliminary prospective, double-blind, placebo-controlled trial. Urology.

[42] Hennenfent BR, Feliciano AE (1998). Changes in white blood cell counts in men undergoing thrice-weekly prostatic massage, microbial diagnosis and antimicrobial therapy for genitourinary complaints. Br J Urol.

[43] Ateya A, Fayez A, Hani R, Zohdy W, Gabbar MA, Shamloul R (2006). Evaluation of prostatic massage in treatment of chronic prostatitis. Urology.

[44] Shoskes DA, Zeitlin SI (1999). Use of prostatic massage in combination with antibiotics in the treatment of chronic prostatitis. Prostate Cancer Prostatic Dis.

[45] Mcnaughton Collins M, MacDonald R, Wilt T (2004). Interventions for chronic abacterial prostatitis (Cochraine Review). The Cochraine library.

[46] Rowe E, Smith C, Laverick L, Elkabir J, Witherow RO, Patel A (2005). A prospective, randomized, placebo controlled, double-blind study of pelvic electromagnetic therapy for the treatment of chronic pelvic pain syndrome with 1 year of follow up. J Urol.

